# Evaluation of Physiological Parameters and Effectiveness of an Immobilization Protocol Using Etorphine, Azaperone, and Butorphanol in Free-Ranging Warthogs (*Phacochoerus africanus*)

**DOI:** 10.3389/fvets.2019.00402

**Published:** 2019-11-14

**Authors:** Donald Neiffer, Peter Buss, Jennie Hewlett, Guy Hausler, Leana Rossouw, Tebogo Manamela, Brittany Grenus, Emily Thulson, Francisco Olea-Popelka, Michele Miller

**Affiliations:** ^1^Wildlife Health Sciences, National Zoological Park, Smithsonian Conservation Biology Institute, Washington, DC, United States; ^2^Veterinary Wildlife Services, South African National Parks, Kruger National Park, Skukuza, South Africa; ^3^Division of Molecular Biology and Human Genetics, Department of Science and Technology-National Research Foundation Centre of Excellence for Biomedical Tuberculosis Research, Faculty of Medicine and Health Sciences, Medical Research Council Centre for Tuberculosis Research, Stellenbosch University, Cape Town, South Africa; ^4^Cummings School of Veterinary Medicine at Tufts University, North Grafton, MA, United States; ^5^Department of Clinical Sciences and Mycobacteria Research Laboratories, College of Veterinary Medicine and Biomedical Science, Colorado State University, Fort Collins, CL, United States

**Keywords:** azaperone, butorphanol, etorphine, immobilization, *Phacochoerus africanus*, warthog

## Abstract

Twenty free-ranging warthogs (*Phacochoerus africanus*) in the Kruger National Park, South Africa, were immobilized with a combination of etorphine (0.039 ± 0.005 mg/kg) and azaperone (0.44 ± 0.06 mg/kg) administered intramuscularly by dart. Butorphanol (1 mg per mg etorphine) was administered intravenously at *t* = 5 min. A standardized scoring system was used to record induction, immobilization and recovery characteristics. Physiological parameters were recorded at 5 min intervals and an arterial sample collected for blood gas analyses every 15 min. At 45 min after butorphanol administration, immobilization was partially reversed by administering naltrexone (40x etorphine dose in mg) intravenously. Overall, induction quality was good, with the mean time to safe handling 5.9 ± 1.4 min. The majority of immobilization scores (54%) over the entire monitoring period (40 min) were at level 3, consistent with a light plane in which palpebral and laryngeal reflexes were still present but the animal could be safely handled. Overall mean heart rate was 94.7 ± 15.3 beats per min, mean respiratory rate was 14.7 ± 9.8 breaths per min, and the mean rectal temperature was 38.5 ± 1.0°C. Significant hypoxia (overall mean oxygen arterial partial pressure 38.8 ± 8.4 mmHg), hypercapnia (mean carbon dioxide arterial partial pressure 63.3 ± 7.8 mmHg), and acidosis (mean pH 7.28 ± 0.04) were observed in immobilized warthogs. Following antagonist administration, warthogs were standing within 1.0 ± 0.4 min, with the majority of recoveries scored as excellent. The drug combination proved to be effective in the immobilization of free-ranging warthogs with rapid induction and recovery, but with significant cardio-respiratory changes. Therefore, this drug combination may be useful when rapid immobilization and recovery are indicated, but should be used cautiously in compromised warthogs.

## Introduction

Warthogs (*Phacochoerus africanus)* are popular inhabitants at zoos and widely distributed in sub-Saharan Africa. However, handling and immobilization can be challenging due to their size, strength and presence of tusks. They can also be difficult to capture due to their acute awareness of their surroundings. Like other suids, they are also susceptible to injury and hyperthermia associated with immobilization (1, 2). Therefore, rapid induction and recovery are important components when choosing an immobilizing drug for these species.

The most common drugs reported for use in warthogs are ketamine (K) and tiletamine-zolazepam (TZ), often combined with medetomidine (M), or azaperone (A) (1, 3). However, these combinations commonly result in falling and paddling during induction and recovery, as well as prolonged periods before animals are recovered sufficiently for release.

Etorphine is a highly potent opioid commonly used for immobilization of wildlife (1). This drug is commonly combined with azaperone to counteract opioid-induced hypertension and lower the dose of etorphine required for immobilization. Etorphine administration results in rapid induction and complete reversibility with naltrexone. However, significant physiological changes including hypoxia, hypercapnia and acidemia are well-documented side effects (4). There are limited reports of etorphine immobilization of warthogs and even less information on physiological responses in this species (2, 5–7). Due to the potential for severe respiratory depression and cardiac arrest, publications that are more recent recommend avoiding its use or completely fail to mention potent opioids as an option for any suid anesthesia (1, 3, 8). This total avoidance of etorphine is not warranted in our experience. Given its availability and since successful immobilization of free-ranging warthogs requires both rapid induction and recovery, etorphine may be a suitable choice, particularly if other drugs can be used to mitigate side effects. Therefore, the aim of this study was to evaluate the effects and suitability of an etorphine, azaperone, butorphanol drug combination in free-ranging warthogs.

## Materials and Methods

The study was conducted in the Kruger National Park (24.9948°S, 31.5969°E) in April 2017. The research protocol was approved by the South African National Parks Animal Use and Care Committee.

Adult warthogs were administered immobilizing drugs into the muscles of the hindquarters using a 3.0 ml plastic dart with 30–40 mm collared needle, propelled by a compressed air dart rifle (DAN-INJECT, International S.A., Skukuza 1350, South Africa). Drug doses were calculated, based on visual estimation of weight, to deliver 0.035 mg/kg etorphine (9.8 mg/ml, M99, Ilanco, Kempton Park 1619, South Africa) and 0.40 mg/kg azaperone (40 mg/ml, Janssen Pharmaceutical Ltd., Halfway House 1685, South Africa). The etorphine dose chosen was based on both experience of the authors and published recommendations (2, 5, 7). After a pilot study, butorphanol (50 mg/ml, Kyron Laboratories, Benrose 2011, South Africa) was added to the immobilization protocol by administering 1:1 butorphanol (mgs) to etorphine (mgs) intravenously 5 min after animal could be safely handled.

Once a warthog was immobilized and safe to handle, it was blindfolded, cotton wool earplugs inserted, and placed in lateral recumbency. Monitoring was started 10 min after initial handling (*t* = 5) to allow repositioning, instrumentation, and butorphanol administration. After induction, study animals were placed in the shade, weighed, and their ears notched for identification to prevent re-darting. At *t* = 45 (~50 min after initial handling), naltrexone (40 mg/ml, Kyron Laboratories), at 40 times the etorphine dose (mgs), was administered into an auricular vein.

Induction times and quality (using a previously described subjective scoring system), immobilization quality, and recovery times and quality were recorded (9). Induction quality was assessed using a scale of 1 (excellent) to 4 (poor). Immobilization depth was scored on a scale of 1–6, with light immobilization equal to a score of 3 (Table 1). Depth was assessed at each 5 min interval during the 40 min monitoring period. Quality of recovery was scored with a 1–4 scale, similar to induction (Table 2).

**Table 1:** Immobilization scoring system for warthogs.

1. Not achieved	Further dosing required for recumbency.
2. Sedated	Responds to manipulation and painful stimulus, voluntary movement, and reflexes present.
3. Light	Safely handled, muscle tone present in jaw and limbs, palpebral, and laryngeal reflexes present.
4. Moderate	No voluntary movement, reduced palpebral and pedal reflexes, relaxed jaw tone, no reaction to sampling.
5. Surgical	Loss of palpebral and pedal reflexes, absent jaw tone, fixed pupils, regular breathing, no reaction to sampling/ear notching.
6. Deep	No reflexes, central miotic pupil, shallow breathing and cardio-respiratory depression.

**Table 2:** Recovery scoring system.

1. Excellent	Sternal score	Antagonist administration to sternal <10 min.
	Standing score	Minimal struggling/stands without falling after minimal attempts.
	Ambulating score	Minimal or no ataxia when walking.
2. Good	Sternal score	Antagonist administration to sternal in 10–20 min.
	Standing score	Transitions to standing with only a few falls before standing successfully.
	Ambulating score	Mild ataxia when walking with few falls.
3. Fair	Sternal score	Antagonist administration to sternal in 20–30 min.
	Standing score	Multiple unsuccessful attempts at standing before able to stand without falling.
	Ambulating score	Moderately ataxic when walking and falls occasionally.
4. Poor	Sternal score	Antagonist administration to sternal >30 min or remained in lateral recumbency.
	Standing score	Unable to stand without falling.
	Ambulating score	Unable to ambulate or falls frequently.

Physiological parameters were recorded at 5 min intervals. Heart rate (HR) was measured by auscultation; respiratory rate (RR) by counting thoracic/abdominal excursions and air movement at the nares; and body temperature (BT) using a digital thermometer placed against the rectal wall. Hemoglobin saturation (SpO_2_) was measured by using a pulse oximeter reflectance probe (TidalGuard SP, SHARN Veterinary Inc., Tampa, Florida 33634, USA) positioned on the conjunctival membranes.

Arterial blood samples, collected from the medial saphenous artery at *t* = 5, *t* = 20, and *t* = 35, were immediately analyzed using a portable blood gas analyzer (iSTAT®1 Handheld Clinical Analyzer, Heska Corporation, Loveland, Colorado 80538, USA) with a CG4+ cartridge (iSTAT CG4+ cartridges, Heska Corporation). Parameters included arterial partial pressure of oxygen (PaO_2_), carbon dioxide (PaCO_2_), pH, lactate, base excess (BE_ecf_), bicarbonate (${\text{HCO}}_{3}^{-}$), and arterial hemoglobin saturation (SaO_2_).

Descriptive statistics (mean, standard deviation, minimum, median, and maximum values) were calculated at different sampling points for induction, immobilization, recovery scores, and physiological and blood gas parameters, using STATA 14 software (Stata Statistical Software, College Station, Texas 77845, USA). Normality of data was assessed by visualizing histograms and using the Shapiro-Wilk test. A linear regression model using ranks was used to evaluate the effect of time (compare values) on the cardiorespiratory and blood gas values including the sample intervals as a fixed effect and using *t* = 5 as the reference values. Non-parametric analysis results were considered statistically significant at *p* < 0.05.

## Results

Twenty free-ranging warthogs (4 males, 16 females), with mean weight of 68.0 kg (range 43.5–105.5 kg), were immobilized using the etorphine, azaperone, and butorphanol drug combination. Mean drug dosages, based on actual body mass, were etorphine 0.039 ± 0.005 mg/kg (range 0.030–0.046 mg/kg), azaperone 0.44 ± 0.06 mg/kg (range 0.35–0.54 mg/kg), and butorphanol 0.39 ± 0.005 mg/kg (range 0.030–0.046 mg/kg).

**Figure 1:**
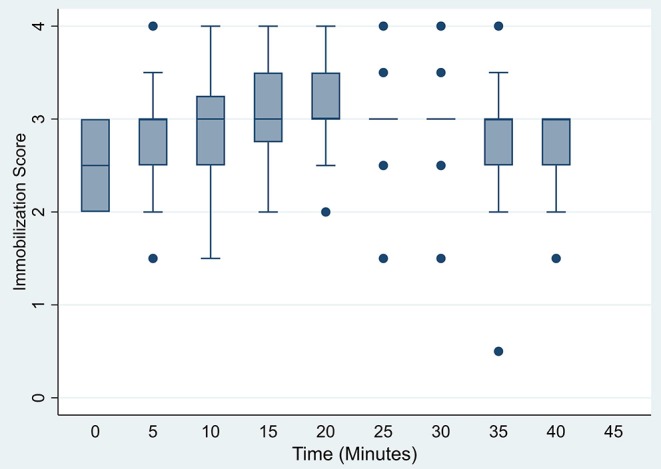
Box plots of immobilization scores for warthogs *(Phacochoerus africanus)* immobilized with etorphine, azaperone, and butorphanol evaluated at 5 min intervals. Horizontal bars represent median values with brackets indicating 95% confidence intervals.

Induction was rapid with ataxia occurring at 2.1 ± 0.5 min (range 1.1–3.0 min). Initial recumbency occurred at 4.8 ± 1.5 min (range 2.4–9.3 min), followed by immobilization (sufficient to allow safe handling) at 5.9 ± 1.4 min (range 3.5–9.6 min). Induction was considered excellent (score 1) in 3 animals, good (score 2) in 16, and fair in 1 warthog of the 20 immobilizations. At initial assessment (*t* = 0), warthogs had immobilization scores of 2–3, with warthogs assessed as sedated to light immobilization (Figure 1). Sedated animals responded to stimuli and had some voluntary movement. Lightly immobilized warthogs maintained some muscle tone and reflexes but could be safely handled. Median immobilization scores increased between *t* = 0 and *t* = 20, with the majority of animals (53–70%) at a light immobilization plane (score 3) between *t* = 20 and *t* = 40. None of the warthogs had an immobilization score of 5 (surgical) or 6 (deep).

**Figure 2:**
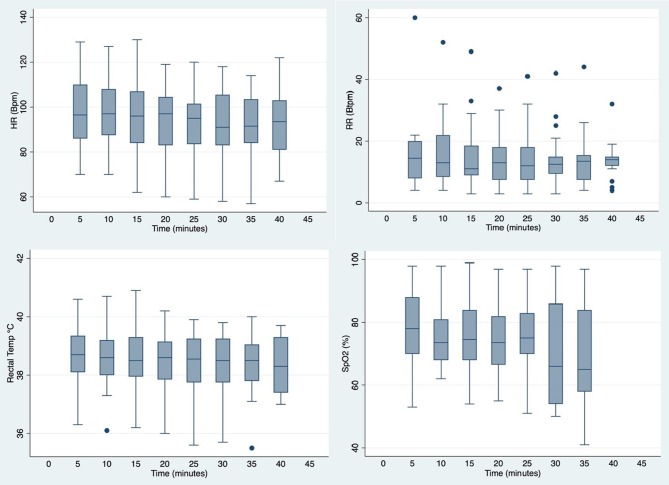
Box plots showing distribution of cardiorespiratory and body temperature values measured at 5 min intervals in warthogs *(Phacochoerus africanus)* immobilized with etorphine, azaperone, and butorphanol. The box represents the interquartile range (25th and 75th percentile) with the horizontal bars inside the box representing median values. The bars represent the lower and upper adjacent values, and outside values are represented by dots.

Figure 2 illustrates cardiorespiratory and BT changes over time (all values are summarized in Supplementary Table 1). Over the 40 min immobilization, there was no significant changes in overall HR, RR and BT. The overall mean HR was 94.7 ± 15.3 beats per min (bpm), with a median of 96 bpm and a range of 57–130 bpm. Overall mean RR was 14.7 ± 9.8 breaths per min (btpm) (median 13 btpm, range 3–60 btpm). Overall mean BT was 38.5 ± 1.0°C (median 38.6°C, range 35.5–40.9°C). The overall mean SpO_2_ value was at 74.0 ± 13.5% (range 41–99%).

**Figure 3:**
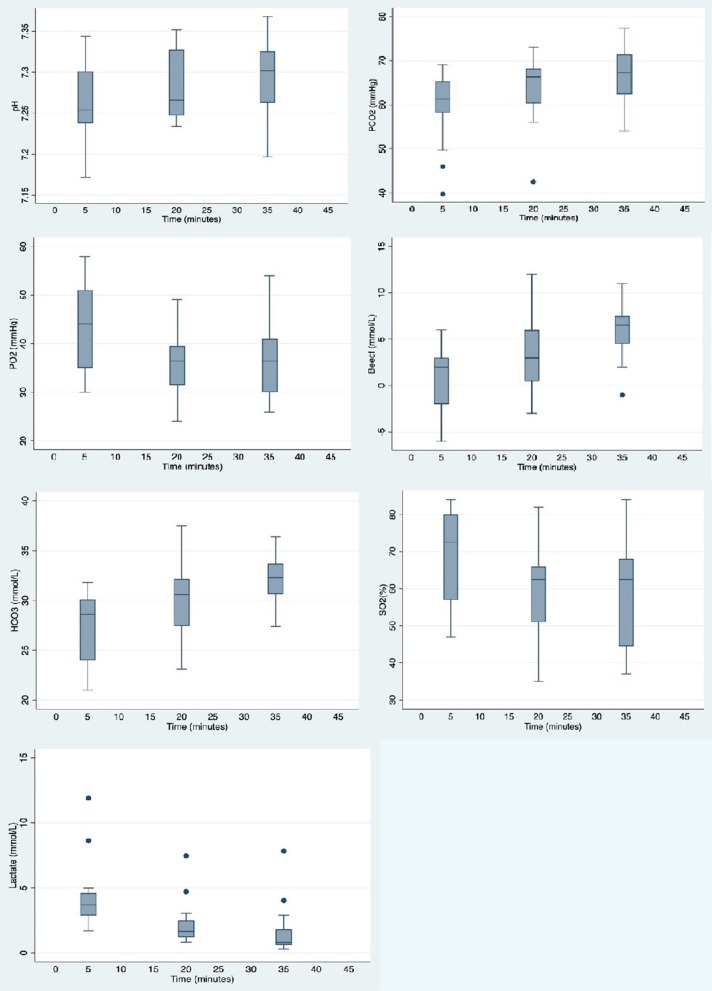
Box plots showing distribution of arterial blood gas values measured at 15 min intervals in warthogs *(Phacochoerus africanus)* immobilized with etorphine, azaperone, and butorphanol. The box represents the interquartile range (25th and 75th percentile) with the horizontal bars inside the box representing median values. The bars represent the lower and upper adjacent values, and outside values are represented by dots.

Figure 3 illustrates arterial blood gas changes over time (all values are summarized in Supplementary Table 2). Warthogs were acidotic with a borderline significant increase in median pH values over time (median pH 7.254 at *t* = 5; 7.302 at *t* = 35; *p* = 0.054). Significant hypoventilation was present at the first time point with median PaCO_2_ and PaO_2_ values of 61.3 mmHg and 44 mmHg, respectively at *t* = 5. These values significantly worsened over time with median values for PaCO_2_ and PaO_2_ at 67.3 and 36.5 mmHg, respectively at *t* = 35 (*p* = 0.045). Median lactate values decreased significantly over time from 3.7 to 0.8 mmol/L between *t* = 5 and *t* = 35 (*p* = 0.001).

Naltrexone was administered to reverse the effects of the etorphine at a mean dosage of 1.64 ± 0.44 mg/kg (range 1.05–3.24 mg/kg). Recovery was rapid with warthogs voluntarily rolling into sternal at a mean time of 0.8 ± 0.4 min (range 0.2–1.8 min) after antidote administration. Sternal recovery was scored as excellent in all but two animals. First attempts to stand occurred at a mean time of 1.0 ± 0.4 min (range 0.2–1.8 min), and successful standing and walking at 1.4 ± 0.5 min (range 0.3–2.1 min). Due to the rapid transition from sternal to standing and walking, the majority of warthogs had a score of 1 for these categories (89, 84, and 89%, respectively). No mortalities or morbidities were observed in any of the immobilized warthogs in this study.

## Discussion

The combination of etorphine, azaperone, and butorphanol administered to free-ranging warthogs consistently produced rapid induction with adequate immobilization to perform minor procedures, including ear notching and arterial blood collection. However, significant hypoventilation with respiratory acidosis was present. In addition, although respiratory rate and body temperature were relatively stable, persistent tachycardia was present for the duration of the immobilization. Recovery was smooth, with rapid controlled transition from recumbency to standing and walking after antidote administration.

Rapid inductions are crucial to immobilizing free-ranging warthogs since they can disappear into the bush after darting. In this study, drug effects were observed by approximately 2 min and warthogs were recumbent and could be handled within 5–6 min. Although a different cohort of warthogs was used, a previous study using free-ranging warthogs in Kruger National Park showed that a combination of medetomidine, butorphanol, tiletamine-zolazepam, and ketamine (MBTZK) resulted in immobilization in a similar time frame (~6 min) (9). These induction times are also similar to those reported using cyclohexylamine and alpha2-agonist immobilization combinations in wild and domestic suids (10–12). Therefore, the use of an opioid immobilization drug combination may not have any advantage over cyclohexylamine-based combinations in terms of faster induction times.

Etorphine can cause significant adverse effects including muscle rigidity, hypertension, and hypoventilation (4, 13). Therefore, etorphine is usually administered in combination with other drugs such as azaperone, alpha2-agonists, butorphanol, or benzodiazepines. In a pilot study, etorphine and azaperone resulted in immobilization in free-ranging warthogs but resulted in severe hypoxia and hypercapnia that required intervention (unpublished data). Since butorphanol administration in etorphine-immobilized white rhinoceros (*Ceratotherium simum*). has been shown to partially reverse these adverse effects, butorphanol was added to the warthog immobilization protocol (4).

Restraint and analgesia are important factors to consider when evaluating immobilization drug combinations. In this study at *t* = 20, 55% of warthogs immobilized with etorphine, azaperone, and butorphanol had reached an immobilization score of 3 (light), with 30% reaching a score of 4 (moderate). Using the same immobilization scoring system to assess responses to stimuli and degree of relaxation, 95% of warthogs immobilized with MBTZK had an immobilization score of 5 (surgical) at *t* = 20 (9). Both drug combinations resulted in a stable immobilization plane for the remaining 20 min of the study. Warthogs administered etorphine, azaperone, and butorphanol never reached a deeper level of immobilization than a score of 4 (moderate–no voluntary movement, reduced palpebral and pedal reflexes, relaxed jaw tone, no reaction to sampling). Most of the observations (54%) were scored at 3, which permitted safe handling, with muscle tone present in jaw and limbs, palpebral and laryngeal reflexes present. In contrast, 79% of total scores for warthogs immobilized with MBTZK were 5, consistent with a surgical plane of anesthesia. These animals had a loss of palpebral and pedal reflexes, absent jaw tone, regular breathing, and no reaction to sampling or ear notching (9). In other studies, warthogs receiving tiletamine-zolazepam and xylazine retained their reflexes (similar to immobilization score 3) (12). However, medetomidine, ketamine, and butorphanol resulted in muscle relaxation, analgesia and loss of reflexes in domestic pigs (similar to immobilization score 4–5) (11). Therefore, the degree of analgesia and restraint required for a procedure will impact the choice of immobilizing drugs.

Tachycardia is commonly observed in animals administered etorphine (13). Although normal values for warthogs are unknown, the mean heart rate values measured in etorphine, azaperone, and butorphanol immobilized animals were considered to be higher than normal with an overall mean of 95 bpm (14). In MBTZK-immobilized warthogs, the overall mean HR was 65 bpm (9). Anesthetized pigs in other studies had reported HR in the range of 70–150 bpm, with bradycardia considered to be <50–70 bpm (14, 15). Butorphanol administration has been shown to significantly reduce overall heart rate in white rhinoceros immobilized with etorphine and azaperone, although still considered tachycardic compared to HR in conscious resting rhinoceros (13). Therefore, although HR appeared elevated in the warthogs, it may be expected that HR would have been higher without the administration of butorphanol.

Respiratory depression is also a common adverse effect of etorphine. In this study, warthogs maintained a stable respiratory rate, with an overall mean value of 15 btpm, during the 40 min monitoring period. In a previous study, MBTZK-immobilized warthogs also had a mean RR of 15 btpm (9). Both of these values were slightly lower than those reported for conscious non-sedated pigs (mean 20, range 16–25 btpm) (14). Higher RR were seen in warthogs anesthetized with a TZ and xylazine combination (range 29 ± 11–32 ± 11 btpm) (12). Although RR appeared to be within acceptable ranges, values for hemoglobin saturation (SpO_2_) and arterial blood gases (PaCO_2_, PaO_2_) were consistent with respiratory depression in the immobilized warthogs in this study.

Pulse oximeters are useful tools for monitoring oxygen saturation in immobilized suids; however, skin or mucous membrane pigmentation can lead to inaccuracy (16). Lower SpO_2_ values have been reported in conscious domestic pigs (mean 94%, range 92–95%), which may be due to species-specific differences in hemoglobin affinity (14). In a previous study, MBTZK-immobilized warthogs had SpO_2_ values between 60 and 100% (9). Similarly, a tiletamine-zolazepam and xylazine combination in warthogs produced SpO_2_ varying between 88 ± 5 and 91 ± 4% (12). Therefore, the low values in etorphine-immobilized warthogs in the current study are likely a true reflection of poor oxygenation. However, in order to determine whether these readings were accurate, blood gas analyses were performed to evaluate ventilation.

In our study, median PaCO_2_ values were elevated at the initial measurement and continued to increase over time (*p* = 0.017). Similarly, low median PaO_2_ values worsened over time (44–36.5 mmHg, *p* = 0.045), which was consistent with significant hypoventilation, likely due to the administration of etorphine (13). In contrast, PaO_2_ values in MBTZK-immobilized warthogs significantly increased over time (median 50 mmHg at *t* = 5–65 mmHg at *t* = 20) (9). However, values for PaO_2_ in conscious domestic pigs ranged from 73 to 92 mmHg, which suggests that the changes may be less severe (14). The low arterial pH in etorphine-immobilized warthogs appeared to be due to respiratory acidosis, consistent with hypoventilation. Values for domestic pigs are reported to be 7.4–7.53 (14). Therefore, significant hypoventilation is a serious adverse effect of the etorphine, azaperone, and butorphanol combination in warthogs, although assisted ventilation could be used to overcome this.

Exertion during induction can exacerbate acidosis and hyperthermia in darted suids. In this study, warthogs had lactate values within normal reported ranges for domestic pigs (0.5–5.5 mmol/L) (17). Body temperatures in this study were also within normal ranges for domestic pigs and provide additional support that the warthogs did not exert themselves post-darting (18). However, these warthogs were habituated to people which may have influenced their response.

Rough recoveries are also a common cause of trauma and hyperthermia in immobilized suids (3, 8). Use of specific reversals for selected immobilizing drugs have been shown in previous studies to improve recovery in suids (10, 11). Administration of atipamezole and naltrexone in MBTZK-immobilized warthogs resulted in rapid smooth recoveries (mean time to walking 7.3 ± 4.9 min) (9). In the current study, the majority of warthogs had excellent recovery scores, with rapid recoveries (mean time to walking <2 min), since naltrexone was used to antagonize the effects of etorphine. The etorphine, azaperone, butorphanol combination may therefore, be especially useful for short procedures in free-ranging warthogs that need to be fully recovered to avoid predation.

Overall, etorphine, azaperone, and butorphanol administration to free-ranging warthogs was an effective immobilizing drug combination with rapid induction and recoveries. However, the adverse cardiorespiratory changes associated with these drugs may increase the risk of morbidity and mortality in compromised warthogs. Therefore, this combination may be useful in free-ranging healthy warthogs for short duration procedures that require rapid restraint and recovery with minimal analgesia, such as snare removal or sampling. This study also provides other alternatives to cyclohexylamine-based combinations for immobilization of this species in zoos.

## Data Availability Statement

The datasets generated for this study are available on request to the corresponding author.

## Ethics Statement

The animal study was reviewed and approved by SAN Parks Animal Use and Care committee.

## Author Contributions

DN, PB, JH, FO-P, and MM responsible for study design, data collection, analyses, and drafting manuscript. GH, LR, TM, BG, ET, and FO-P participated in data collection and analyses. ET and FO-P were responsible for statistical calculations. All authors assisted with writing and editing the manuscript.

### Conflict of Interest

The authors declare that the research was conducted in the absence of any commercial or financial relationships that could be construed as a potential conflict of interest.
