# Supplementary material for: Evaluation of Physiological Parameters and Effectiveness of an Immobilization Protocol Using Etorphine, Azaperone, and Butorphanol in Free-Ranging Warthogs (Phacochoerus africanus)
Source: Front Vet Sci. 2019 Nov 14;6:402. doi: 10.3389/fvets.2019.00402 (PMC6867999; doi:10.3389/fvets.2019.00402)
Supplement: Supplementary file 1 [file Table_1.pdf]

Supplementary Table 1. Distribution of cardiorespiratory and body temperature values measured at 5 min intervals in warthogs immobilized with etorphine, azaperone, and butorphanol.

Time (min)	Statistics	HR (bpm)	RR (btpm)	SPO2 (%)	Temperature (°C)
5	Mean	98.7	15.9	76.5	38.7
	SD	15.3	12	13.3	1
	Min	70	4	53	36.3
	Q1	86	8	70	38.1
	Median	96.5	14.5	78	38.7
	Q3	110	20	88	39.3
	Max	129	60	98	40.6
	n	20	20	19	20
10	Mean	98.1	15.7	76.3	38.5
	SD	14.8	11.7	11.4	1
	Min	70	4	62	36.1
	Q1	87.5	8.5	68	38
	Median	97	13	73.5	38.6
	Q3	108	22	81	39.2
	Max	127	52	98	40.7
	n	20	20	18	20
15	Mean	96	15.1	76.5	38.6
	SD	16.6	11.4	12.7	1.1
	Min	62	3	54	36.2
	Q1	84	9	68	37.9
	Median	96	11	74.5	38.5
	Q3	107	18.5	84	39.3
	Max	130	49	99	40.9
	n	20	20	20	20
20	Mean	94.1	14.3	74.3	38.5
	SD	15	9.4	11	1
	Min	60	3	55	36
	Q1	83	7.5	66.5	37.8
	Median	97	13	73.5	38.6
	Q3	104.5	18	82	39.1
	Max	119	37	97	40.2
	n	20	20	20	20
25	Mean	93.1	14.3	75.9	38.4
	SD	15.5	9.5	13.1	1
	Min	59	3	51	35.6
	Q1	83.5	7.5	70	37.7
	Median	95	12	75	38.5
	Q3	101.5	18	83	39.2
	Max	120	41	97	39.9
	n	20	20	17	20
30	Mean	92.5	14.1	70.5	38.4
	SD	16.1	9.1	15.8	1

	Min	58	3	50	35.7
	Q1	83	9.5	54	37.7
	Median	91	12.5	66	38.5
	Q3	105.5	15	86	39.2
	Max	118	42	98	39.8
	n	20	20	18	20
35	Mean	91.8	14.4	68	38.3
	SD	14.2	9	16.4	1
	Min	57	4	41	35.5
	Q1	84	7.5	58	37.8
	Median	91.5	13.5	65	38.5
	Q3	103.5	15.5	84	39
	Max	114	44	97	40
	n	20	20	18	20
40	Mean	93.2	14	n.a.	38.3
	SD	15.4	6.2	n.a.	0.9
	Min	67	4	n.a.	37
	Q1	81	12	n.a.	37.4
	Median	93.5	14	n.a.	38.3
	Q3	103	15	n.a.	39.3
	Max	122	32	n.a.	39.7
	n	18	17	0	10
Total	Mean	94.7	14.7	74.1	38.5
	SD	15.2	9.8	13.5	1
	Min	57	3	41	35.5
	Q1	84	8	65	38
	Median	96	13	73	38.5
	Q3	105	17	84	39.3
	Max	130	60	99	40.9
	n	158	157	130	150

n.a. – not available
