# Supplementary material for: Evaluation of Physiological Parameters and Effectiveness of an Immobilization Protocol Using Etorphine, Azaperone, and Butorphanol in Free-Ranging Warthogs (Phacochoerus africanus)
Source: Front Vet Sci. 2019 Nov 14;6:402. doi: 10.3389/fvets.2019.00402 (PMC6867999; doi:10.3389/fvets.2019.00402)
Supplement: Supplementary file 2 [file Table_2.pdf]

Supplementary Table 2. Distribution of arterial blood gas values measured at 15 minute intervals in warthogs immobilized with etorphine, azaperone, and butorphanol.

Time (min)	Statistics	pH	PCO_2 (mm hg)	PO_2 (mm hg)	Base Excess (mmol/L)	HCO_3 (mm Hg)	SO_2 (%)	Lactate (mmol/L)
5	Mean	7.266	59.8	43.2	0.6	27.2	68.4	4.29
	SD	0.043	8.2	9.3	3.9	3.7	13.1	2.57
	Min	7.172	39.7	30	-6	21	47	1.69
	Q1	7.238	58.2	35	-2	24	57	2.87
	Median	7.254	61.3	44	2	28.6	72.5	3.68
	Q3	7.301	65.3	51	3	30.1	80	4.6
	Max	7.344	69.1	58	6	31.8	84	11.9
	n	17	17	16	17	17	16	16
20	Mean	7.282	64	36.2	3.5	30.2	59.1	2.21
	SD	0.041	7.4	6	3.9	3.5	11.4	1.7
	Min	7.234	42.5	24	-3	23.1	35	0.82
	Q1	7.247	60.3	31.5	0.5	27.4	51	1.21
	Median	7.266	66.3	36.5	3	30.6	62.5	1.65
	Q3	7.327	68.1	39.5	6	32.1	66	2.49
	Max	7.352	73.1	49	12	37.5	82	7.46
	n	16	16	16	16	16	16	16
35	Mean	7.296	66.3	36.9	5.9	32.2	59.6	1.65
	SD	0.045	6.7	8.2	2.8	2.3	15.6	1.93
	Min	7.197	54	26	-1	27.4	37	0.3
	Q1	7.263	62.3	30	4.5	30.6	44.5	0.63
	Median	7.302	67.3	36.5	6.5	32.3	62.5	0.81
	Q3	7.325	71.4	41	7.5	33.7	68	1.82
	Max	7.368	77.3	54	11	36.4	84	7.83
	n	16	16	16	16	16	16	16
Total	Mean	7.281	63.3	38.8	3.3	29.8	62.3	2.71
	SD	0.044	7.8	8.4	4.1	3.8	13.9	2.35
	Min	7.172	39.7	24	-6	21	35	0.3
	Q1	7.246	60	31.5	1	28.1	51	1.09
	Median	7.277	64.8	37.5	4	30.3	63	1.94
	Q3	7.316	68	44	6	31.8	72.5	3.68
	Max	7.368	77.3	58	12	37.5	84	11.9
	n	49	49	48	49	49	48	48
